# Extracellular Matrix Protein Ratios in the Human Heart and Vessels: How to Distinguish Pathological From Physiological Changes?

**DOI:** 10.3389/fphys.2021.708656

**Published:** 2021-08-04

**Authors:** Corey Wittig, Robert Szulcek

**Affiliations:** Laboratory of in vitro Modeling Systems of Pulmonary Diseases, Institute of Physiology, Charité – Universitätsmedizin Berlin, Corporate Member of Freie Universität Berlin and Humboldt-Universität zu Berlin, Berlin, Germany

**Keywords:** cardiovascular, elastin, human, extracellular matrix, pathology, heart, aorta, collagen

## Abstract

Cardiovascular pathology is often accompanied by changes in relative content and/or ratios of structural extracellular matrix (ECM) proteins within the heart and elastic vessels. Three of these proteins, collagen-I, collagen-III, and elastin, make up the bulk of the ECM proteins in these tissues, forming a microenvironment that strongly dictates the tissue biomechanical properties and effectiveness of cardiac and vascular function. In this review, we aim to elucidate how the ratios of collagen-I to collagen-III and elastin to collagen are altered in cardiovascular diseases and the aged individuum. We elaborate on these major cardiovascular ECM proteins in terms of structure, tissue localization, turnover, and physiological function and address how their ratios change in aging, dilated cardiomyopathy, coronary artery disease with myocardial infarction, atrial fibrillation, aortic aneurysms, atherosclerosis, and hypertension. To the end of guiding *in vitro* modeling approaches, we focus our review on the human heart and aorta, discuss limitations in ECM protein quantification methodology, examine comparability between studies, and highlight potential *in vitro* applications. In summary, we found collagen-I relative concentration to increase or stay the same in cardiovascular disease, resulting in a tendency for increased collagen-I/collagen-III and decreased elastin/collagen ratios. These ratios were found to fall on a continuous scale with ranges defining distinct pathological states as well as a significant difference between the human heart and aortic ECM protein ratios.

## Introduction

The extracellular matrix (ECM) is a highly dynamic, biologically active, non-cellular mesh of fibrous proteins, glycoproteins, and glycosaminoglycans that is secreted by many cell types and is present within all tissues. The ECM provides active biochemical and biomechanical cues for a vast range of cellular functions, serves as a physical scaffold, and a cell growth substrate. ECM proteins can be categorized as either structural (stable) or non-structural (dynamic), of which the structural proteins generate mechanical and biophysical properties of tissues (Frantz et al., 2010). Collagen-I (COL1) and III (COL3), as well as elastin (ELN), are the most prominent ECM structural proteins of the cardiovascular system. These proteins have a key role in modulating the intricate balance of elasticity, resilience, and rigidity that is necessary for physiological function, given the dynamic and distinct contractile and elastic nature of the heart and aorta.

In cardiovascular pathology, the ratios of these ECM proteins are frequently shifted with disease progression. With this change, cardiovascular function is altered in terms of mechanical performance, as well as the biophysical signaling of the microenvironment. In this review, we specify COL1/COL3, and elastin to total collagen (ELN/COL) ratio-changes in multiple cardiovascular diseases and aging. We focus our analyses strictly on human data based on the significant observed variation in these ratios between species, identifying a knowledge gap in recent literature and providing a translatable ECM protein ratio reference for *in vitro* microenvironmental modeling of human physiology and pathology.

## Major Cardiovascular Extracellular Matrix Proteins

### Collagen

One of the major structural proteins of the ECM, collagen is widely distributed extracellularly in most tissues, of which COL1 and COL3 are two of the most common types (von der Mark, 1981). COL1 is a triple-helical fibril protein consisting of two pro-α1(I)-chains and one pro-α2(I)-chain and forms into dense, thick rod-like bundles that organize as multiple cross-linked rows of parallel fibers. COL3 is a homotrimer of three pro-α1(III)-chains and typically forms more loosely-packed bundles of thin fibrils also into cross-linked rows (Gelse et al., 2003). While both COL1 and COL3 fibril diameter can vary between 25 and 80 nm, cardiac COL1 fibril diameters average at 75 nm, while COL3 diameters average at 45 nm (de Souza, 2002).

COL1 and COL3 are both predominately secreted by fibroblasts and smooth muscle cells. While COL1 is the major collagen type present in bone, tendons, dermis, ligaments, and connective tissues, COL3 is distributed mainly in the skin, vessel walls, and reticular fibers of most tissues (Goel et al., 2013). Furthermore, COL1 and COL3 are the co-principal forms in the myocardium (von der Mark, 1981). Collagen in general displays a low synthesis rate but longer half-life, estimated at 80–120 days in the heart and 60–70 days in vessels (Nissen et al., 1978; Weber, 1989).

Both fibrillar collagens serve similar physiological functions both mechanically and with shared α_1_β_1_/α_2_β_1_ integrin- and DDR1/DDR2 receptor-binding sites, controlling mechanical properties and directly regulating factors, such as cell migration, proliferation, and tissue homeostasis, through binding-mediated intracellular signaling (Sainio and Järveläinen, 2020). However, COL1 fibrils are notably stiffer and provide structural rigidity and strength, torsional stability, and the coordination of delivery of force in certain niches, such as the myocardium, thus reducing tissue compliance (Gelse et al., 2003). COL3 fibrils are thinner and impart less tensile strength than COL1, but are more elastic, thus providing more resilience, tissue distensibility, and structural maintenance in expansion. Combined with the collagen type ratios, alignment, configuration, and extent of crosslinking, the mechanical and behavioral properties of tissues are modulated (Weber, 1989; de Souza, 2002).

### Elastin

Elastin is an extensible, structural protein distributed in the cardiovascular system, connective tissue, lungs, and skin and is known to be highly durable due to its extensive crosslinking and hydrophobicity (Wise and Weiss, 2009; Xu and Shi, 2014). Elastin is composed of single tropoelastin subunits cross-linked with an outer layer of fibrillin microfibers to form an elastic fiber. Tropoelastin conveys the ability of the fiber to stretch and recoil, while the fibrillin microfibers contribute to fiber rigidity (Wise and Weiss, 2009).

Elastin is secreted by fibroblasts and smooth muscle cells and is a major ECM component in elastic arterial and venous vessel walls (Xu and Shi, 2014). It has minimal turnover and a very long half-life of 40–70 years with gene expression and protein synthesis almost exclusively occurring in the late embryonic stage (Xu and Shi, 2014; Cocciolone et al., 2018).

As elastin is over three orders of magnitude less stiff than collagen, elastin does not significantly contribute to ECM structural rigidity or strength (Dobrin, 1978). Furthermore, it is unknown how tropoelastin binding to integrin α_V_β_3_ modulates intracellular signaling, although elastin degradation products (elastokines) are known to bind to the elastin receptor complex, inducing protease expression and secretion, angiogenesis, and cell migration, adhesion, survival, and proliferation (Bax et al., 2009; Scandolera et al., 2016). The most prominent physiologic function of elastin, however, is to permit elasticity and recoil from stretch. Due to extensive crosslinking and hydrophobicity, elastin is also the most durable ECM element, providing integrity against degradation from repeated stretch-recoil cycles (Wise and Weiss, 2009).

### Protein Quantification Methods

Multiple methods exist to isolate and separate ECM protein types for quantification, falling into two categories: biochemical digestion analysis or staining. While biochemical analysis allows for direct quantification of collagen, it is hampered by incomplete protein separation. Quantitative staining allows regional visualization and quantification of all proteins present within a single slice but fails to account for variations in protein content within the slice thickness by only providing 2D area-based quantification.

Quantitative imaging through quantitative immunohistochemistry, histochemistry, and immunofluorescence are frequently used for both collagen and elastin. Relative protein quantification of these stained samples is done either visually under a microscope as percent area stained or using microdensitophotometry (van der Loos et al., 1994).

In the widely-used cyanogen bromide (CNBr) cleavage biochemical technique, pepsin digestion is followed by cleavage with CNBr to peptides varying in molecular weight and unique for each collagen type (separation efficiency: >90%). These can then be separated and quantified through electrophoresis, followed by spectrophotometry/densitometry/chromatography (Kaiser and Metzka, 1999). Differential salt precipitation also allows for the quantification of specific collagen types (separation efficiency: 84–96%) but relies on multiple separation stages (Rhodes and Miller, 1978). When only total collagen is measured, hydroxyproline (a major collagen component) is measured and multiplied by a conversion factor to determine total collagen (extraction efficiency: >98%; Neuman and Logan, 1950; Jackson and Cleary, 1967; Stegemann and Stalder, 1967).

Biochemical elastin quantification techniques are also used, with CNBr incubation and acetone washing used to break cross-links and isolate elastin (Starcher and Galione, 1976). However, the dominant biochemical method is the hot alkali method (NaOH digestion at 100°C) to extract elastin although there is evidence of elastin degradation (Lansing et al., 1952). Measurement of desmosine and isodesmosine (elastin amino acids), and subsequent calculation of elastin content is another technique (Volpin and Michelotto, 1973). Efficiency of elastin extraction in these methods appears to be similar and high though there is a possibility for noncollagenous protein contamination (Soskel and Sandburg, 1983).

## Pathophysiological Relationship Between Structural Extracellular Matrix Proteins

### Collagen-I/Collagen-III Ratio

**Figure 1:**
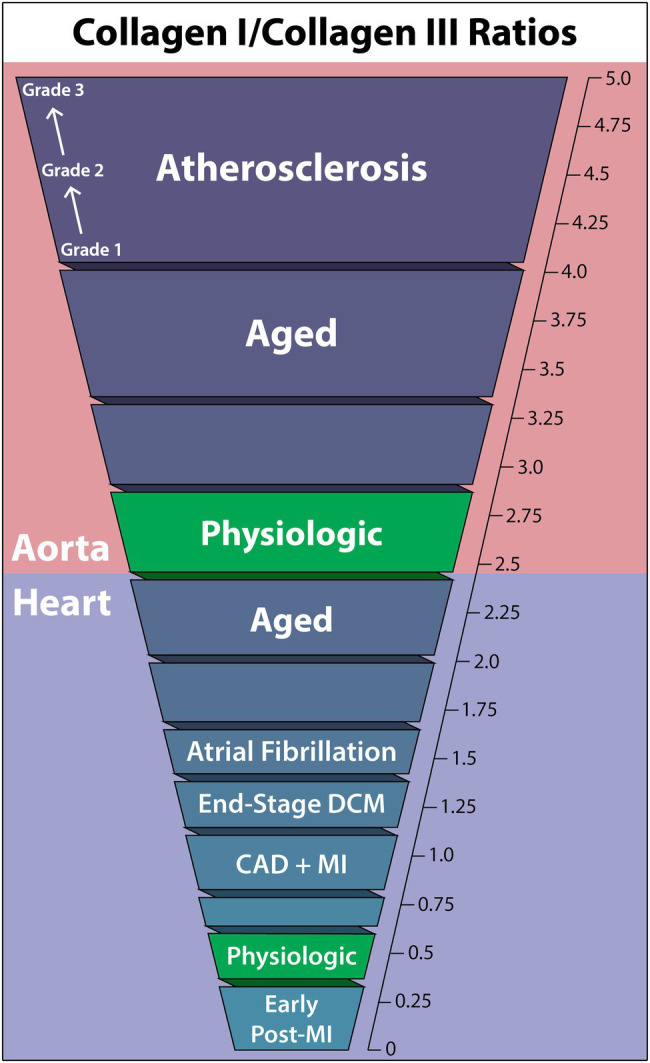
Collagen-I to collagen-III ratios within the human aorta and heart in cardiovascular diseases, the aged individuum, and the physiologic situation. Collagen-I increases relative to collagen-III in all non-physiologic cases other than early post-myocardial infarction, and the aorta is seen to have a much higher collagen-I/collagen-III ratio than the heart in general. DCM, dilated cardiomyopathy; CAD, coronary artery disease; MI, myocardial infarction.

The relative ratios of collagen types are heavily dependent on the tissue and organ, varying considerably between the heart and aorta. This ratio, which helps to define particular pathological states, can fluctuate in both directions. Alterations in this ratio in the course of cardiovascular disease may severely impact physiological function as tissue mechanical properties change. For instance, biomarker data suggested that an increase in the COL1/COL3 ratio occurs in hypertensive heart disease, while the opposite has been suggested in chamber dilatation (Collier et al., 2012). Furthermore, correlations have been found between collagen type expression ratios and left ventricular (LV) function (Soufen et al., 2008; Polyakova et al., 2011). The following COL1/COL3 ratio findings are summarized in Figure 1.

#### Heart

##### Physiologic

In healthy humans, a typical COL1/COL3 ratio of the LV has been consistently observed to fall within 0.3–0.6, depending on the method of quantification (Marijianowski et al., 1995, 1997; Pauschinger et al., 1999; Polyakova et al., 2011). This predominance of COL3 is in direct contrast to cardiac collagen content in other species, where the myocardium of healthy long-tailed macaques contained 85% COL1 and 11% COL3 (Weber et al., 1988), rat myocardium COL1/COL3 ratio was 6.41 (Mukherjee and Sen, 1993), and porcine myocardium showed a COL1/COL3 ratio of 2.3 (Medugorac, 1982). As factors, such as heart rate, lifestyle, vessel size, etc., contribute to the resulting forces placed on the heart, species-dependent cardiovascular physiological differences likely are key in determining the ideal tensile strength and elasticity of the heart, thereby accounting for species ratio differences and emphasizing the necessity of human-specific data.

##### Aged

While the heart is known to undergo collagen ratio changes with age, the degree of change has yet to be investigated quantitatively (Schwach and Passier, 2019). Nevertheless, two histochemistry studies have demonstrated the propensity of the aged heart to develop an increased COL1/COL3 ratio, in which stained COL1 increases and COL3 decreases to almost negligible levels with age. This, coupled with the observation that fibril diameter consistently increased from youth to elderly, suggests a major increase in the COL1/COL3 ratio in the aged heart (Debessa et al., 2001; Mendes et al., 2012).

##### Dilated Cardiomyopathy

End-stage dilated cardiomyopathy (DCM) shows a clear trend toward elevated COL1 in the heart. Pauschinger investigated protein levels by quantitative immunohistochemistry, finding interstitial fibrosis and increased COL1/COL3 from 0.33 in controls (*n* = 18) to 0.66 in end-stage DCM (*n* = 12) in the right ventricle (Pauschinger et al., 1999). A similar result using quantitative immunofluorescence was obtained by Polyakova, who found mainly interstitial fibrosis, some perivascular fibrosis, and a COL1/COL3 ratio increase from 0.30 in controls (*n* = 8) to 0.53 in end-stage DCM (*n* = 6) in the LV (Polyakova et al., 2011).

Investigations into COL1/COL3 quantification of the human LV in end-stage DCM have also been performed using CNBr cleavage, providing improved accuracy. Generally, the same trend was found with an increasing COL1/COL3 ratio from healthy controls to DCM patients: 0.60 (*n* = 16) to 1.27 (*n* = 19) in 20 μm-thickness myocardium samples (Marijianowski et al., 1995) and 1.39 (*n* = 25) to 1.89 (*n* = 5) in full-thickness myocardium samples (Bishop et al., 1990).

##### Coronary Artery Disease With Myocardial Infarction

Coronary artery disease (CAD) with associated myocardial infarction (MI) has been observed to have a regional and transient change in COL1/COL3 ratios. Circulating biomarkers were used to identify increased COL3 synthesis immediately post-MI that is sustained for 1 year, decreasing the COL1/COL3 ratio. This trend begins to reverse 2 months post-MI, once increased COL1 synthesis begins (Manhenke et al., 2014).

Quantitative immunofluorescence identified an increase in the COL1/COL3 ratio from 0.3 in controls to 1.0 in LV tissue bordering the infarct area, along with significant replacement fibrosis. In “remote” regions not near infarcts, perivascular and interstitial fibrosis was dominant, though a non-significant COL1/COL3 ratio-change from 0.3 (*n* = 8) to 0.4 (*n* = 7) was observed (Polyakova et al., 2011). Additional quantitative immunohistochemistry studies using 6 μm-thickness myocardium samples reached similar conclusions, finding that non-scarred areas had no COL1/COL3 ratio increase, while scar-border areas revealed a ratio increase from 0.54 in controls (*n* = 16) to 0.87 in CAD (*n* = 15; Marijianowski et al., 1997). Bishop reported very high values in full-thickness CNBr-digested myocardium samples with comparable relative ratio-changes from 1.39 in controls (*n* = 25) to 1.79 in CAD (*n* = 10) but did not control for location of tissue sampling relative to infarcts (Bishop et al., 1990).

##### Atrial Fibrillation

Atrial fibrillation (AF) COL1/COL3 ratios have twice been quantified in the left atrium. Boldt utilized Western blot to determine that both lone AF and mitral valve disease with AF are associated with interstitial fibrosis through an increased COL1/COL3 ratio in the left atrium from 0.97 in healthy controls (*n* = 16) to 1.55 in AF (*n* = 86; Boldt et al., 2004). This change was exclusively due to COL1 increase, as COL3 levels remained the same between controls and patients. This result has been supported by findings showing increased COL1 circulating biomarkers in AF prevalence, incidence, and recurrence although no direct tissue sampling was done (Ravassa et al., 2019). However, another quantitative study of COL1/COL3 ratio-changes in AF found no significant collagen differences in the left atrium due to AF (Smorodinova et al., 2015).

##### Hypertension

No quantitative study has yet been performed on human hearts that measures both collagen-I and COL3 in hypertension. However, one qualitative study examined the LV myocardium from adults with essential hypertension for ≥5 years, finding a general, unquantified increase in both COL1 and III (Mindán and Panizo, 1993).

Multiple rat models have been used to quantify COL1/COL3 changes in the hypertensive heart, although no conclusive trend is seen as different studies present a CO1/COL3 ratio increase, decrease, and location-specific change (Mukherjee and Sen, 1990; Robert et al., 1994). One human circulating biomarker study found increased COL3 synthesis and altered COL1 turnover favoring degradation in symptomatic heart failure essential hypertension patients, suggesting a decreased COL1/COL3 ratio, although the location of collagen turnover is indeterminable in circulating biomarker studies (Plaksej et al., 2009).

#### Aorta

##### Physiologic

In the aorta, the normal physiologic range of the COL1/COL3 ratio is somewhat unclear, with measured values ranging from 2.04 to 3.83, with only minor deviation based on location or layer (intima vs. media vs. complete vessel wall; Murata et al., 1986; Rizzo et al., 1989; Sobolewski et al., 1995). However, the typical finding suggests an approximate COL1/COL3 range between 2.4 and 2.8.

##### Aged

Like in the heart, collagen concentration in vessels is generally accepted to increase with age. However, how the ratio of COL1/COL3 changes is less understood (Schlatmann and Becker, 1977; Miller et al., 1993; Cattell et al., 1996). In a study grouping patients by age [<50 (*n* = 11) and >50 (*n* = 11)] and using CNBr cleavage, the aortic arch COL1/COL3 ratio was noted to increase from 3.0 to 3.37, while the lower abdominal aorta saw an increase from 2.47 to 4.05 (Maurel et al., 1987). Therefore, age-related changes in the aortic COL1/COL3 ratio seem to be location-dependent but generally increasing.

##### Aortic Aneurysms

Abdominal aortic aneurysms (AAA) are understood to be associated with alterations in collagen content of the human aorta (Bode et al., 2000; Treska and Topolčan, 2000; Wilson et al., 2001; Carmo et al., 2002). However, in two independent studies, the ratio of COL1/COL3 was insignificantly changed in AAA aortic walls as compared to healthy controls, with ratio-changes of (healthy-to-AAA) 2.7 (*n* = 13) to 2.8 (*n* = 19) using CNBr cleavage (Rizzo et al., 1989) and 2.7 (*n* = 7) to 2.2 (*n* = 13) using differential salt precipitation (Sobolewski et al., 1995).

##### Atherosclerosis

In atherosclerosis, collagen type distribution within atherosclerotic plaques significantly deviates from healthy aortic walls, especially in advanced cases (McCullagh et al., 1980). By sampling undiseased and graded 1–3 atherosclerotic tissue from the aortic intima and using differential salt precipitation for collagen type quantification, it was observed that only COL1 amount changed relative to undiseased tissue, increasing the COL1/COL3 ratio with atherosclerosis grade from 3.83 (*n* = 16) in undiseased to 4.88 in grade 3 (*n* = 5; Murata et al., 1986). Furthermore, Morton showed that thoracic and abdominal aorta atherosclerotic plaques COL1/COL3 ratio increased from 2.04 (*n* = 6) in non-atherosclerotic aortic intima layers to 2.48 in plaques (*n* = 7), using CNBr cleavage (Morton and Barnes, 1982).

##### Hypertension

While direct COL1/COL3 ratio changes due to hypertension have not yet been quantified in human aorta studies, animal models have demonstrated no significant changes in the COL1/COL3 ratio in the hypertensive aorta (Clerc et al., 1999). However, a biomarker-based collagen turnover study on arterial hypertension patients found a significant alteration in collagen turnover favoring increased COL1 accumulation although COL3 synthesis was also observed to be increased (Stakos et al., 2010). Thus, how the ratio of these two proteins changes in hypertensive humans remains unknown.

### Elastin/Collagen Ratio

**Figure 2:**
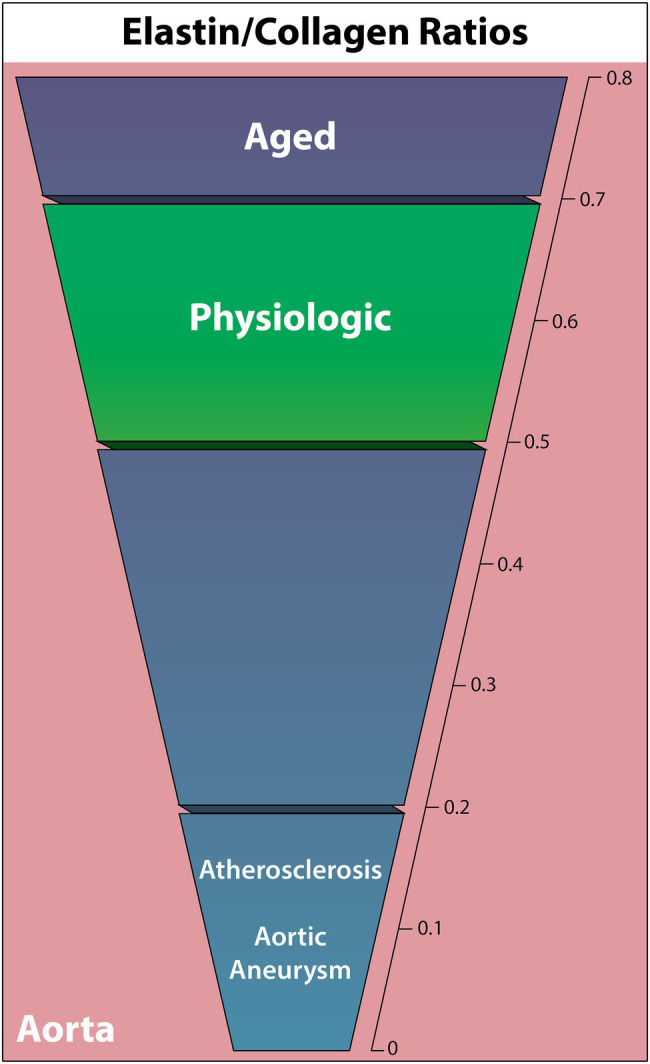
Elastin to collagen ratios in the human complete aortic wall in cardiovascular diseases, the aged individuum, and the physiologic situation. Elastin relative to collagen is noted to decrease in all non-physiologic cases other than aging.

Elastic arteries utilize significantly more elastic tissue in the tunica media than muscular arteries, enabling a reversible stretch response to maintain a relatively constant pressure gradient (Cocciolone et al., 2018). Based on this function, alterations in the mechanical properties of elastic arteries are commonly associated with cardiovascular disease as the arteries adapt to or fail to properly deform under hemodynamic load. As resistance to stretch at low pressures is due to elastic fibers, and resistance to stretch at high pressures is due to collagen fibers, the ratio of these two components is vital to understanding the ECM contributions to cardiovascular pathology (Cocciolone et al., 2018). The following ELN/COL ratio findings are summarized in Figure 2.

#### Aorta

##### Physiologic

Relative elastin and collagen content is known to vary depending on artery, distance from the heart, and vessel layer (Cattell et al., 1996; Sokolis et al., 2012). However, a comprehensive quantitative human elastic vessel ECM protein study that covers all these variables has not yet been performed. In a full aortic wall, a normal middle-aged physiologic ELN/COL ratio appears to be consistently between 0.5 and 0.7 (Rizzo et al., 1989; Sobolewski et al., 1995; Cattell et al., 1996). Meanwhile, the isolated intima and intima-media have higher ELN/COL ratios around 1.7–1.9 (Rokosova et al., 1986; Iliopoulos et al., 2009).

##### Aged

Highly durable against degradation, ELN is known to have essentially no synthesis following adolescence (Cocciolone et al., 2018). This, coupled with multiple studies showing decreased aortic compliance with age, suggests a declining ELN/COL ratio with age (Sonesson et al., 1993; Bulpitt et al., 1999; McVeigh et al., 1999; Gardner and Parker, 2010).

Interestingly, the most recent aging study in 20 normotensive thoracic aortic walls found that, while both collagen (hydroxyproline analysis) and elastin (CNBr digestion) absolute content decreases with age, they both increase in concentration relative to tissue dry weight with age (Cattell et al., 1996). This results in an increased ELN/COL ratio from 0.51 at age 14, to 0.6 at age 40, and 0.71 at age 90, opposing the expected ELN/COL ratio decline with age. In contrast, Andreotti found the opposite trend in a study of 22 thoracic aortas aged 9–84, where collagen concentration (hydroxyproline analysis) increased with age and ELN concentration (hot alkali extraction) decreased with age, thus resulting in a decreased ELN/COL ratio, although exact elastin quantification was not given (Andreotti et al., 1985). A decreasing trend in the ELN/COL ratio in 137 ascending aortas, from 1.65 in ages 0–20 to 1.15 in ages 70–100 was observed in another study using hot alkali extraction for elastin and hydroxyproline analysis for collagen. However, the change was nonsignificant (Hosoda et al., 1984).

##### Aortic Aneurysms

Elastin quantification following AAA and ascending thoracic aortic aneurysms (ATAA) has demonstrated ELN degradation in a number of cases (Campa et al., 1987; Baxter et al., 1992; Wilson et al., 2001; Carmo et al., 2002). The ELN/COL ratio-changes were first quantified in aortic walls using quantitative histochemistry, observing a significant ratio decrease from 0.41 in nonaneurysmal abdominal aortas (*n* = 8) to 0.02 in AAA (*n* = 8; He and Roach, 1994). This was predominately due to minimal elastin in AAA (89.4% decrease), although collagen also increased by 77%. Rizzo found a similar substantial increase in collagen (hydroxyproline analysis) and decrease in elastin (hot alkali extraction) to only 1% in AAA aortic walls, reducing the ELN/COL ratio from 0.5 (*n* = 13) to 0.03 (*n* = 19; Rizzo et al., 1989). Similarly, multiple other studies identified a trend of decreasing ELN/COL ratio and significantly fewer ELN crosslinks in AAA patients, as the ELN/COL ratio decreased in nonruptured AAA from 0.63 (*n* = 7) to 0.2 (*n* = 13; Sobolewski et al., 1995) and 0.84 (*n* = 24) to 0.13 (*n* = 26; Carmo et al., 2002).

Comparable ELN/COL changes were observed in ATAA with age-matched human aortas, sampled both orientationally and within each layer of the aortic wall (Iliopoulos et al., 2009; Sokolis et al., 2012). Using quantitative histochemistry, the ELA/COL ratio was found to decrease in only the intima of the aorta, finding healthy (*n* = 60) to ATAA (*n* = 104) ratios of 0.74 to 0.52 in the entire aortic wall, 1.74 to 0.83 in the intima, 0.84 to 0.81 in the media, and 0.24 to 0.24 in the adventitia.

##### Atherosclerosis

Studies with parallel elastin and collagen quantification in human atherosclerotic aortas are scarce. Nevertheless, Rokosova has demonstrated that advanced atherosclerotic lesions in the distal aorta against the intima-media layers of healthy aortae (controls were significantly younger) show significantly more collagen (hydroxyproline analysis) and less elastin (desmosine analysis), decreasing the healthy-diseased ELN/COL ratio from 1.88 (*n* = 14) to 0.14 (*n* = 22; Rokosova et al., 1986).

This result is supported by findings of decreased ELN in plaques compared to normal adjacent aortic intima in all three grades of atherosclerosis and significantly decreased soluble elastin in the core of lipid-rich and ruptured plaques (though not in fiber-rich plaques) compared to healthy aortic intima tissue (Kramsch et al., 1971; Akima et al., 2009). As stated previously, collagen is also known to increase in atherosclerotic plaques, supporting a decreased ELN/COL ratio (Partridge and Keeley, 1974; Murata et al., 1986).

##### Hypertension

Only one human hypertensive aorta study from 1952 measured both elastin and collagen, comparing on an age-related basis using the whole intima and media (Faber and Møller-Hou, 1952). Here, it was observed that hypertensive and normal aortas had relatively similar collagen and elastin content at most ages, although at later ages (≥60), hypertensive aortas tended to have both increased collagen and decreased elastin compared to nonhypertensive similarly-aged aortas although significance is not noted. Additionally, numerous animal models support a decreased ELN/COL ratio in hypertension (see Arribas et al., 2006 for review).

## Conclusion

The major cardiovascular ECM proteins, COL1, COL3, and ELN each have a critical role in maintaining proper cardiovascular function, modulating the structural and elastic properties of the heart and major elastic vessels. As seen in this review, a number of attempts have been made to quantify ratio-changes within humans in a range of cardiovascular diseases, aiding in the determination of how the biomechanics of the heart and aorta may shift in the course of disease progression.

Generally, COL1 relative concentration was always observed to increase or stay the same in cardiovascular disease. This aligns with the commonly observed stiffening of both the heart and aorta in many cardiovascular diseases that are often characterized by an increased hemodynamic load. Thus, the trend followed that COL1/COL3 increased while the ELN/COL ratios decreased, although the degree of change varied considerably, especially when comparing the heart and aorta, where the aorta was found to have considerably higher COL1/COL3 ratios. This is possibly due to the different dilation vs. contraction demands and forces subjected to the aorta and heart, where aortic COL1 may be necessary in higher amounts to provide structural integrity, prevent breakage, and reduce vascular wall susceptibility to fatigue and failure in the presence of high relative elastin content (Vouyouka et al., 2001). Furthermore, as low myocardial compliance contributes to diastolic dysfunction through ventricular hypertrophy and associated reduced ventricular filling due to reduced expansion capacity, COL3 may be more necessary to provide structural maintenance while also permitting distensibility and contraction following expansion (Du et al., 2003).

Interestingly, these ratios were found to fall on a continuous scale that defines physiological and distinct pathological states, with clear distinctions between heart and vessel. Unfortunately, as demonstrated in this review, COL1/COL3 and ELN/COL ratios in the human heart and aorta remain minimally researched or severely-outdated in literature, leaving a considerable knowledge gap for even some of the most widespread cardiovascular diseases. The reported protein ratios provide crucial information into the patho(physio)logical microenvironment experienced by the cells and offer a way to recreate the major biophysical properties of the substrate in 2D-cell-cultures through coatings that employ the relative ratios discussed in this review. While such a strategy would clearly not capture a large majority of the ECM properties and signaling present *in vivo*, an enhanced ratio-based culture coating offers an ECM that more accurately replicates the bulk of the tissue dry weight protein content, supporting pathological cell sensitivities, morphologies, and behaviors that may only emerge/are more apparent in certain biophysical conditions or by particular extracellular ligand binding.

While a number of human studies were identified in this review, several methodological factors limit the cross-comparison both between and within these studies. While most investigations employed CNBr cleavage to isolate the proteins, different methodologies with different sensitivities were used, including hot alkali extraction, differential salt precipitation, quantitative immunohistochemistry, and more, potentially confounding the relational ability between studies due to different technique efficiencies. However, even when identical protein isolation and separation techniques were used, significant differences were occasionally noted in determined ratios. This was clear in DCM, where two studies using CNBr cleavage found highly different COL1/COL3 myocardial ratios, while the only methodological difference was the thickness of the sampled tissue, implying regional COL1/COL3 differences even within the isolated myocardium. Furthermore, due to limited human sample availability, sample numbers were often low (typically <20 samples per study) and exhibited high sample-to-sample variation. Lastly, high variability in both age and gender that skews toward elderly males in diseased samples as opposed to lower ages in physiologic control samples may further cloud the exact ratio-changes in disease.

In summary, we review human-specific quantitative measurements of major structural ECM proteins (COL1, COL3, and ELN) in the heart and aorta. We determine how the COL1/COL3 and ELN/COL ratios deviate from physiologic baselines in cardiovascular diseases and the aged individuum and identify a continuous scale defining physiological and distinct pathological stages. Using these ratios for *in vitro* disease modeling provides a straightforward method to more accurately capture the patho(physio)logical biophysical microenvironment in a number of diseases, potentially enhancing cell pathophenotype *in vitro*.

## Author Contributions

CW wrote the manuscript. CW and RS created the figures, revised the manuscript, and conceptualized the topic. RS provided the funding. All authors contributed to the article and approved the submitted version.

## Conflict of Interest

The authors declare that the research was conducted in the absence of any commercial or financial relationships that could be construed as a potential conflict of interest.

## Publisher’s Note

All claims expressed in this article are solely those of the authors and do not necessarily represent those of their affiliated organizations, or those of the publisher, the editors and the reviewers. Any product that may be evaluated in this article, or claim that may be made by its manufacturer, is not guaranteed or endorsed by the publisher.

## References

[ref1] Akima T. Nakanishi K. Suzuki K. Katayama M. Ohsuzu F. Kawai T. (2009). Soluble elastin decreases in the progress of atheroma formation in human aorta. Circ. J. 73, 2154–2162. 10.1253/circj.CJ-09-0104, PMID: 19755752

[ref2] Andreotti L. Bussotti A. Cammelli D. di Giovine F. Sampognaro S. Sterrantino G.. (1985). Aortic connective tissue in ageing—a biochemical study. Angiology 36, 872–879. 10.1177/000331978503601206, PMID: 4083569

[ref3] Arribas S. M. Hinek A. González M. C. (2006). Elastic fibres and vascular structure in hypertension. Pharmacol. Ther. 111, 771–791. 10.1016/j.pharmthera.2005.12.003, PMID: 16488477

[ref4] Bax D. V. Rodgers U. R. Bilek M. M. M. Weiss A. S. (2009). Cell adhesion to tropoelastin is mediated via the C-terminal GRKRK motif and integrin αVβ3. J. Biol. Chem. 284, 28616–28623. 10.1074/jbc.M109.017525, PMID: 19617625 PMC2781405

[ref5] Baxter B. T. McGee G. S. Shively V. P. Drummond I. A. S. Dixit S. N. Yamauchi M.. (1992). Elastin content, cross-links, and mRNA in normal and aneurysmal human aorta. J. Vasc. Surg. 16, 192–200. 10.1016/0741-5214(92)90107-J, PMID: 1495142

[ref6] Bishop J. E. Greenbaum R. Gibson D. G. Yacoub M. Laurent G. J. (1990). Enhanced deposition of predominantly type I collagen in myocardial disease. J. Mol. Cell. Cardiol. 22, 1157–1165. 10.1016/0022-2828(90)90079-H, PMID: 2095438

[ref7] Bode M. K. Soini Y. Melkko J. Satta J. Risteli L. Risteli J. (2000). Increased amount of type III pN-collagen in human abdominal aortic aneurysms: evidence for impaired type III collagen fibrillogenesis. J. Vasc. Surg. 32, 1201–1207. 10.1067/mva.2000.109743, PMID: 11107093

[ref8] Boldt A. Wetzel U. Lauschke J. Weigl J. Gummert J. Hindricks G.. (2004). Fibrosis in left atrial tissue of patients with atrial fibrillation with and without underlying mitral valve disease. Heart 90, 400–405. 10.1136/hrt.2003.015347, PMID: 15020515 PMC1768173

[ref9] Bulpitt C. J. Rajkumar C. Cameron J. D. (1999). Vascular compliance as a measure of biological age. J. Am. Geriatr. Soc. 47, 657–663. 10.1111/j.1532-5415.1999.tb01586.x, PMID: 10366163

[ref10] Campa J. S. Greenhalgh R. M. Powell J. T. (1987). Elastin degradation in abdominal aortic aneurysms. Atherosclerosis 65, 13–21. 10.1016/0021-9150(87)90003-7, PMID: 3649236

[ref11] Carmo M. Colombo L. Bruno A. Corsi F. R. M. Roncoroni L. Cuttin M. S.. (2002). Alteration of elastin, collagen and their cross-links in abdominal aortic aneurysms. Eur. J. Vasc. Endovasc. Surg. 23, 543–549. 10.1053/ejvs.2002.1620, PMID: 12093072

[ref12] Cattell M. A. Anderson J. C. Hasleton P. S. (1996). Age-related changes in amounts and concentrations of collagen and elastin in normotensive human thoracic aorta. Clin. Chim. Acta 245, 73–84. 10.1016/0009-8981(95)06174-6, PMID: 8646817

[ref13] Clerc P. C. Renaud J. F. Blacher J. Legrand M. Samuel J. L. Levy B. I.. (1999). Collagen I and III and mechanical properties of conduit arteries in rats with genetic hypertension. J. Vasc. Res. 36, 139–146. 10.1159/000025637, PMID: 10213910

[ref14] Cocciolone A. J. Hawes J. Z. Staiculescu M. C. Johnson E. O. Murshed M. Wagenseil J. E. (2018). Elastin, arterial mechanics, and cardiovascular disease. Am. J. Physiol. Heart Circ. Physiol. 315, H189–H205. 10.1152/ajpheart.00087.2018, PMID: 29631368 PMC6139627

[ref15] Collier P. Watson C. J. van Es M. H. Phelan D. McGorrian C. Tolan M.. (2012). Getting to the heart of cardiac remodeling; how collagen subtypes may contribute to phenotype. J. Mol. Cell. Cardiol. 52, 148–153. 10.1016/j.yjmcc.2011.10.002, PMID: 22008391

[ref17] Debessa C. R. G. Maifrino L. B. M. de Souza R. R. (2001). Age related changes of the collagen network of the human heart. Mech. Ageing Dev. 122, 1049–1058. 10.1016/S0047-6374(01)00238-X, PMID: 11389923

[ref16] de Souza R. R. (2002). Aging of myocardial collagen. Biogerontology 3, 325–335. 10.1023/A:1021312027486, PMID: 12510171

[ref18] Dobrin P. B. (1978). Mechanical properties of arteries. Physiol. Rev. 58, 397–460. 10.1152/physrev.1978.58.2.397, PMID: 347471

[ref19] Du X.-J. Samuel C. S. Gao X.-M. Zhao L. Parry L. J. (2003). Increased myocardial collagen and ventricular diastolic dysfunction in relaxin deficient mice: a gender-specific phenotype. Cardiovasc. Res. 57, 395–404. 10.1016/S0008-6363(02)00663-6, PMID: 12566112

[ref20] Faber M. Møller-Hou G. (1952). The human aorta. APMIS 31, 377–382. 10.1111/j.1699-0463.1952.tb00205.x 12985344

[ref21] Frantz C. Stewart K. M. Weaver V. M. (2010). The extracellular matrix at a glance. J. Cell Sci. 123, 4195–4200. 10.1242/jcs.023820, PMID: 21123617 PMC2995612

[ref22] Gardner A. W. Parker D. E. (2010). Association between arterial compliance and age in participants 9 to 77 years old. Angiology 61, 37–41. 10.1177/0003319709339588, PMID: 19638351 PMC2798009

[ref23] Gelse K. Pöschl E. Aigner T. (2003). Collagens – structure, function, and biosynthesis. Adv. Drug Deliv. Rev. 55, 1531–1546. 10.1016/j.addr.2003.08.002, PMID: 14623400

[ref24] Goel S. A. Guo L. W. Shi X. D. Kundi R. Sovinski G. Seedial S.. (2013). Preferential secretion of collagen type 3 versus type 1 from adventitial fibroblasts stimulated by TGF-β/Smad3-treated medial smooth muscle cells. Cell. Signal. 25, 955–960. 10.1016/j.cellsig.2012.12.021, PMID: 23280188 PMC3595331

[ref25] He C. M. Roach M. R. (1994). The composition and mechanical properties of abdominal aortic aneurysms. J. Vasc. Surg. 20, 6–13. 10.1016/0741-5214(94)90169-4, PMID: 8028090

[ref26] Hosoda Y. Kawano K. Yamasawa F. Ishii T. Shibata T. Inayama S. (1984). Age-dependent changes of collagen and elastin content in human aorta and pulmonary artery. Angiology 35, 615–621. 10.1177/000331978403501001, PMID: 6497045

[ref27] Iliopoulos D. C. Kritharis E. P. Giagini A. T. Papadodima S. A. Sokolis D. P. (2009). Ascending thoracic aortic aneurysms are associated with compositional remodeling and vessel stiffening but not weakening in age-matched subjects. J. Thorac. Cardiovasc. Surg. 137, 101–109. 10.1016/j.jtcvs.2008.07.023, PMID: 19154911

[ref28] Jackson D. S. Cleary E. G. (1967). The determination of collagen and elastin. Methods Biochem. Anal. 15, 25–76. 10.1002/9780470110331.ch2, PMID: 4899619

[ref29] Kaiser R. Metzka L. (1999). Enhancement of cyanogen bromide cleavage yields for methionyl-serine and methionyl-threonine peptide bonds. Anal. Biochem. 266, 1–8. 10.1006/abio.1998.2945, PMID: 9887207

[ref30] Kramsch D. M. Franzblau C. Hollander W. (1971). The protein and lipid composition of arterial elastin and its relationship to lipid accumulation in the atherosclerotic plaque. J. Clin. Invest. 50, 1666–1677. 10.1172/JCI106656, PMID: 5097573 PMC442067

[ref31] Lansing A. I. Rosenthal T. B. Alex M. Dempsey E. W. (1952). The structure and chemical characterization of elastic fibers as reveled by elastase and by electron microscopy. Anat. Rec. 114, 555–575. 10.1002/ar.1091140404, PMID: 13016985

[ref32] Manhenke C. Ueland T. Jugdutt B. I. Godang K. Aukrust P. Dickstein K.. (2014). The relationship between markers of extracellular cardiac matrix turnover: infarct healing and left ventricular remodelling following primary PCI in patients with first-time STEMI. Eur. Heart J. 35, 395–402. 10.1093/eurheartj/eht482, PMID: 24255130

[ref33] Marijianowski M. M. H. Teeling P. Becker A. E. (1997). Remodeling after myocardial infarction in humans is not associated with interstitial fibrosis of noninfarcted myocardium. J. Am. Coll. Cardiol. 30, 76–82. 10.1016/S0735-1097(97)00100-9, PMID: 9207624

[ref34] Marijianowski M. M. H. Teeling P. Mann J. Becker A. E. (1995). Dilated cardiomyopathy is associated with an increase in the type I/type III collagen ratio: a quantitative assessment. J. Am. Coll. Cardiol. 25, 1263–1272. 10.1016/0735-1097(94)00557-7, PMID: 7722119

[ref35] Maurel E. Shuttleworth C. A. Bouissou H. (1987). Interstitial collagens and ageing in human aorta. Virchows Arch. A Pathol. Anat. Histopathol. 410, 383–390. 10.1007/BF00712757, PMID: 3103320

[ref36] McCullagh K. G. Duance V. C. Bishop K. A. (1980). The distribution of collagen types I, III and V (AB) in normal and atherosclerotic human aorta. J. Pathol. 130, 45–55. 10.1002/path.1711300107, PMID: 6991657

[ref37] McVeigh G. E. Bratteli C. W. Morgan D. J. Alinder C. M. Glasser S. P. Finkelstein S. M.. (1999). Age-related abnormalities in arterial compliance identified by pressure pulse contour analysis: aging and arterial compliance. Hypertension 33, 1392–1398. 10.1161/01.HYP.33.6.1392, PMID: 10373222

[ref38] Medugorac I. (1982). Collagen type distribution in the mammalian left ventricle during growth and aging. Res. Exp. Med. 180, 255–262. 10.1007/BF01852298, PMID: 7123010

[ref39] Mendes A. B. L. Ferro M. Rodrigues B. de Souza M. R. Araujo R. C. de Souza R. R. (2012). Quantification of left ventricular myocardial collagen system in children, young adults, and the elderly. Medicina 72, 216–220. PMID: 22763158

[ref40] Miller E. J. Malcom G. T. McMahan C. A. Strong J. P. (1993). Atherosclerosis in young white males: arterial collagen and cholesterol. Matrix 13, 289–296. 10.1016/S0934-8832(11)80024-7, PMID: 8412986

[ref41] Mindán F. Panizo A. (1993). Alterations in the extracellular matrix of the myocardium in essential hypertension. Eur. Heart J. 14(Suppl. J), 12–14. PMID: 8281955

[ref42] Morton L. F. Barnes M. J. (1982). Collagen polymorphism in the normal and diseased blood vessel wall. Investigation of collagens types I, III and V. Atherosclerosis 42, 41–51. 10.1016/0021-9150(82)90124-1, PMID: 7082417

[ref43] Mukherjee D. Sen S. (1990). Collagen phenotypes during development and regression of myocardial hypertrophy in spontaneously hypertensive rats. Circ. Res. 67, 1474–1480. 10.1161/01.RES.67.6.1474, PMID: 2147130

[ref44] Mukherjee D. Sen S. (1993). Alteration of cardiac collagen phenotypes in hypertensive hypertrophy: role of blood pressure. J. Mol. Cell. Cardiol. 25, 185–196. 10.1006/jmcc.1993.1021, PMID: 8474126

[ref45] Murata K. Motayama T. Kotake C. (1986). Collagen types in various layers of the human aorta and their changes with the atherosclerotic process. Atherosclerosis 60, 251–262. 10.1016/0021-9150(86)90172-3, PMID: 3089234

[ref46] Neuman R. E. Logan M. A. (1950). The determination of hydroxyproline. J. Biol. Chem. 184, 299–306. 10.1016/S0021-9258(19)51149-8, PMID: 15421999

[ref47] Nissen R. Cardinale G. J. Udenfriend S. (1978). Increased turnover of arterial collagen in hypertensive rats. Proc. Natl. Acad. Sci. U. S. A. 75, 451–453. 10.1073/pnas.75.1.451, PMID: 272662 PMC411267

[ref48] Partridge S. M. Keeley F. W. (1974). “Age related and atherosclerotic changes in aortic elastin,” in Arterial Mesenchyme and Arteriosclerosis. eds. Wagner W. Clarkson T. (Boston, MA: Springer), 173–191. 10.1007/978-1-4684-3243-5_9 4599722

[ref49] Pauschinger M. Knopf D. Petschauer S. Doerner A. Poller W. Schwimmbeck P. L.. (1999). Dilated cardiomyopathy is associated with significant changes in collagen type I/III ratio. Circulation 99, 2750–2756. 10.1161/01.CIR.99.21.2750, PMID: 10351968

[ref50] Plaksej R. Kosmala W. Frantz S. Herrmann S. Niemann M. Störk S.. (2009). Relation of circulating markers of fibrosis and progression of left and right ventricular dysfunction in hypertensive patients with heart failure. J. Hypertens. 27, 2483–2491. 10.1097/HJH.0b013e3283316c4d, PMID: 19887955

[ref51] Polyakova V. Loeffler I. Hein S. Miyagawa S. Piotrowska I. Dammer S.. (2011). Fibrosis in endstage human heart failure: severe changes in collagen metabolism and MMP/TIMP profiles. Int. J. Cardiol. 151, 18–33. 10.1016/j.ijcard.2010.04.053, PMID: 20546954

[ref52] Ravassa S. Ballesteros G. López B. Ramos P. Bragard J. González A.. (2019). Combination of circulating type i collagen-related biomarkers is associated with atrial fibrillation. J. Am. Coll. Cardiol. 73, 1398–1410. 10.1016/j.jacc.2018.12.074, PMID: 30922470

[ref53] Rhodes R. K. Miller E. J. (1978). Physicochemical characterization and molecular organization of the collagen A and B chains. Biochemistry 17, 3442–3448. 10.1021/bi00610a003, PMID: 687595

[ref54] Rizzo R. J. McCarthy W. J. Dixit S. N. Lilly M. P. Shively V. P. Flinn W. R.. (1989). Collagen types and matrix protein content in human abdominal aortic aneurysms. J. Vasc. Surg. 10, 365–373. 10.1016/0741-5214(89)90409-6, PMID: 2795760

[ref55] Robert V. van Thiem N. Cheav S. L. Mouas C. Swynghedauw B. Delcayre C. (1994). Increased cardiac types I and III collagen mRNAs in aldosterone-salt hypertension. Hypertension 24, 30–36. 10.1161/01.HYP.24.1.30, PMID: 8021005

[ref56] Rokosova B. Rapp J. H. Porter J. M. Peter Bentley J. (1986). Composition and metabolism of symptomatic distal aortic plaque. J. Vasc. Surg. 3, 617–622. 10.1016/0741-5214(86)90286-7, PMID: 3959257

[ref57] Sainio A. Järveläinen H. (2020). Extracellular matrix-cell interactions: focus on therapeutic applications. Cell. Signal. 66:109487. 10.1016/j.cellsig.2019.109487, PMID: 31778739

[ref58] Scandolera A. Odoul L. Salesse S. Guillot A. Blaise S. Kawecki C.. (2016). The elastin receptor complex: a unique matricellular receptor with high anti-tumoral potential. Front. Pharmacol. 7:32. 10.3389/fphar.2016.00032, PMID: 26973522 PMC4777733

[ref59] Schlatmann T. J. M. Becker A. E. (1977). Histologic changes in the normal aging aorta: implications for dissecting aortic aneurysm. Am. J. Cardiol. 39, 13–20. 10.1016/S0002-9149(77)80004-0, PMID: 831420

[ref60] Schwach V. Passier R. (2019). Native cardiac environment and its impact on engineering cardiac tissue. Biomater. Sci. 7, 3566–3580. 10.1039/C8BM01348A, PMID: 31338495

[ref61] Smorodinova N. Lantová L. Bláha M. Melenovský V. Hanzelka J. Pirk J.. (2015). Bioptic study of left and right atrial interstitium in cardiac patients with and without atrial fibrillation: Interatrial but not rhythm-based differences. PLoS One 10:e0129124. 10.1371/journal.pone.0129124, PMID: 26067062 PMC4466374

[ref62] Sobolewski K. Wolańska M. Bańkowski E. Gacko M. Głowiński S. (1995). Collagen, elastin and glycosaminoglycans in aortic aneurysms. Acta Biochim. Pol. 42, 301–307. 10.18388/abp.1995_4588, PMID: 8588480

[ref63] Sokolis D. P. Krithais E. P. Giagini A. T. Lampropoulos K. M. Papadodima S. A. Iliopoulos D. C. (2012). Biomechanical response of ascending thoracic aortic aneurysms: association with structural remodelling. Comput. Methods Biomech. Biomed. Engin. 15, 231–248. 10.1080/10255842.2010.522186, PMID: 21480082

[ref64] Sonesson B. Hansen F. Stale H. Länne T. (1993). Compliance and diameter in the human abdominal aorta—the influence of age and sex. Eur. J. Vasc. Surg. 7, 690–697. 10.1016/S0950-821X(05)80718-2, PMID: 8270073

[ref65] Soskel N. T. Sandburg L. B. (1983). A comparison of six methods of extracting elastin residue from hamster lungs. Exp. Lung Res. 4, 109–119. 10.3109/01902148309055008, PMID: 6840044

[ref66] Soufen H. N. Salemi V. M. C. Aneas I. M. S. Ramires F. J. A. Benício A. M. D. Benvenuti L. A.. (2008). Collagen content, but not the ratios of collagen type III/I mRNAs, differs among hypertensive, alcoholic, and idiopathic dilated cardiomyopathy. Braz. J. Med. Biol. Res. 41, 1098–1104. 10.1590/S0100-879X2008001200009, PMID: 19148372

[ref67] Stakos D. A. Tziakas D. N. Chalikias G. K. Mitrousi K. Tsigalou C. Boudoulas H. (2010). Associations between collagen synthesis and degradation and aortic function in arterial hypertension. Am. J. Hypertens. 23, 488–494. 10.1038/ajh.2010.2, PMID: 20134406

[ref68] Starcher B. C. Galione M. J. (1976). Purification and comparison of elastins from different animal species. Anal. Biochem. 74, 441–447. 10.1016/0003-2697(76)90224-4, PMID: 822746

[ref69] Stegemann H. Stalder K. (1967). Determination of hydroxyproline. Clin. Chim. Acta 18, 267–273. 10.1016/0009-8981(67)90167-2, PMID: 4864804

[ref70] Treska V. Topolčan O. (2000). Plasma and tissue levels of collagen types I and III markers in patients with abdominal aortic aneurysms. Int. Angiol. 19, 64–68. PMID: 10853688

[ref71] van der Loos C. M. Marijianowski M. M. H. Becker A. E. (1994). Quantification in immunohistochemistry: the measurement of the ratios of collagen types I and III. Histochem. J. 26, 347–354. 10.1007/BF00157768, PMID: 8040007

[ref72] Volpin D. Michelotto G. (1973). Procedure for the automatic analysis of all amino acids in elastin hydrolyzates on a routine basis. J. Chromatogr. A 79, 335–336. 10.1016/S0021-9673(01)85307-3, PMID: 4736158

[ref73] von der Mark K. (1981). Localization of collagen types in tissues. Int. Rev. Connect. Tissue Res. 9, 265–324. 10.1016/b978-0-12-363709-3.50012-7, PMID: 6175597

[ref74] Vouyouka A. G. Pfeiffer B. J. Liem T. K. Taylor T. A. Mudaliar J. Phillips C. L. (2001). The role of type I collagen in aortic wall strength with a homotrimeric [α1(I)]3 collagen mouse model. J. Vasc. Surg. 33, 1263–1270. 10.1067/mva.2001.113579, PMID: 11389427

[ref75] Weber K. T. (1989). Cardiac interstitium in health and disease: the fibrillar collagen network. J. Am. Coll. Cardiol. 13, 1637–1652. 10.1016/0735-1097(89)90360-4, PMID: 2656824

[ref76] Weber K. T. Janicki J. S. Shroff S. G. Pick R. Chen R. M. Bashey R. I. (1988). Collagen remodeling of the pressure-overloaded, hypertrophied nonhuman primate myocardium. Circ. Res. 62, 757–765. 10.1161/01.RES.62.4.757, PMID: 2964945

[ref77] Wilson K. A. Lindholt J. S. Hoskins P. R. Heickendorff L. Vammen S. Bradbury A. W. (2001). The relationship between abdominal aortic aneurysm distensibility and serum markers of elastin and collagen metabolism. Eur. J. Vasc. Endovasc. Surg. 21, 175–178. 10.1053/ejvs.2001.1303, PMID: 11237793

[ref78] Wise S. G. Weiss A. S. (2009). Tropoelastin. Int. J. Biochem. Cell Biol. 41, 494–497. 10.1016/j.biocel.2008.03.017, PMID: 18468477

[ref79] Xu J. Shi G. P. (2014). Vascular wall extracellular matrix proteins and vascular diseases. Biochim. Biophys. Acta Mol. Basis Dis. 1842, 2106–2119. 10.1016/j.bbadis.2014.07.008, PMID: 25045854 PMC4188798

